# Supplementary material for: Fecal glucocorticoid metabolite levels in captive Indian leopards (Panthera pardus fusca) housed under three different enrichment regimes
Source: PLoS One. 2022 Sep 9;17(9):e0261796. doi: 10.1371/journal.pone.0261796 (PMC9462577; doi:10.1371/journal.pone.0261796)
Supplement: S1 Table — (DOCX) [file pone.0261796.s001.docx]

**Supplemental Information**

| Indian leopards (our data) | | Indian leopards (Vaz *et al.* 2017) | | African leopards  (Webster *et al.* 2018) | | Remarks |
| --- | --- | --- | --- | --- | --- | --- |
| Captivity | Wild | Captivity | Wild | Captivity | Wild | Webster *et al.* and our data used the same fGCM assay |
| 10.40±3.87, 0.2-108.63 | 0.96±0.16, 0.2-1.93 | 0.27±0.013 | NA | 0.29-0.39 | 0.02-6.09 |  |

**Table S1** Comparative overview of fecal glucocorticoid metabolite (fGCM) levels (Mean±SEM), and value range; µg/g dry feces) in Indian leopards from our data and in Indian and African leopards from published data.
