# Supplementary material for: Fecal glucocorticoid metabolite levels in captive Indian leopards (Panthera pardus fusca) housed under three different enrichment regimes
Source: PLoS One. 2022 Sep 9;17(9):e0261796. doi: 10.1371/journal.pone.0261796 (PMC9462577; doi:10.1371/journal.pone.0261796)
Supplement: Attachment — Submitted filename: PlosRev(RG)21stJan22.docx [file pone.0261796.s003.docx]

In the manuscript titled, “Effect of active and passive enrichment regimes on fecal glucocorticoid metabolite (fGCM) levels in captive Indian leopards”, authors have measured fGCM levels of captive Indian leopards, housed in three types of housing environments with varied enrichments and, showed that fGCM levels in leopards housed in active enrichment type with small or medium size cages without or with air coolers were significantly different and that housing animals with active enrichment element influence physiological well-being of captive leopards. A number of appropriate statistical analyse were performed to validate the data and conclusions drawn. The study outcomes provide improvising opportunities in the zoo management practices, focusing on categories of animals being housed, coupled with environment specific enrichment. These findings are important in the context of strategic development of captive management of big cats, Indian leopards, with implications in their conservation.

*Comments:*

1. Abstract section: specify values of fGCM levels in categories B and C (lines 25-26). Also, replace “informing” by “informed” (line 29).
2. It is unclear as to what could have been the age of free-ranging animals (n = 12) while collecting opportunistic scat samples. Time elapsed of scat sampling, post-defecation, of free-ranging animals is unclear. This could be a variable factor, while comparing the fGCMs, unlike fresh samples collected from captive animals in zoos.
3. Scat samples of zoo animals were collected in distinctly at two different (summer and winter) seasons. However, it is unclear as to why the scat samples of free ranging animals were collected at different times (Jan-Mar), unlike the month duration for scat sampling of captive animals during two seasons.
4. What is the GCM extraction efficiency; any recovery experiment performed with scat samples.
5. Line 247: sensitivity – “0.8 pg” indicated is per well or per ml or per gm dry weight?
6. Line 248: A comma is missing at “respectively”.
7. The last conclusion-paragraph (lines 453-472) should be concise. Redundant statements (for example, ‘enrichment’ and ‘management programmes/policies’) would need to be eliminated.
