# Supplementary material for: Fecal glucocorticoid metabolite levels in captive Indian leopards (Panthera pardus fusca) housed under three different enrichment regimes
Source: PLoS One. 2022 Sep 9;17(9):e0261796. doi: 10.1371/journal.pone.0261796 (PMC9462577; doi:10.1371/journal.pone.0261796)
Supplement: Attachment — Submitted filename: Response to reviewers.docx [file pone.0261796.s004.docx]

**Journal requirements:**

1. *Please ensure that your manuscript meets PLOS ONE's style requirements, including those for file naming.*

Response: We have ensured that the manuscript meets the style requirements as	prescribed by the journal.

2. *We suggest you thoroughly copyedit your manuscript for language usage, spelling, and grammar. If you do not know anyone who can help you do this, you may wish to consider employing a professional scientific editing service.*

Response: We have now substantially revised the manuscript as per the	suggestions of the three reviewers, and have also edited the revised version for	improving the language and for addressing the grammatical errors.

3. *We note that the grant information you provided in the ‘Funding Information’ and ‘Financial Disclosure’ sections do not match.*

Response: We have now provided the correct statement for both the ‘Funding	information’ and ‘Financial disclosure’ sections.

4. *Please remove any funding-related text from the manuscript and let us know how you would like to update your Funding Statement.*

Response: We have revised the data availability statement and removed the	funding related text from the main body of the manuscript.

5. *In your Data Availability statement, you have not specified where the minimal data set underlying the results described in your manuscript can be found.*

Response: We would like to confirm that all the data pertaining to the manuscript	is available within the results, figures and supplementary sections of the revised	version of the	manuscript. We have updated the same in the Data Availability	statement.

6. *We note that you have included the phrase “data not shown” in your manuscript. Unfortunately, this does not meet our data sharing requirements.*

Response: We apologize for including the phrase. We would like to confirm that	all the data pertaining to the manuscript is available within the results, figures	and supplementary sections of the revised version of the manuscript.

7. *We note that Figure 1 in your submission contain map images which may be copyrighted.*

Response: With regard to Figure 1, we have obtained the raster map from the	USGS site ([http://eros.usgs.gov/#](http://eros.usgs.gov/)), which according to Plos One policy	(<https://journals.plos.org/plosone/s/figures#loc-licenses-and-copyright>) is	compatible with CC by licensing. We sincerely hope that the revised manuscript	addresses all the concerns raised by the Journal.

8. *Please include captions for your Supporting Information files at the end of your manuscript, and update any in-text citations to match accordingly*

Response: We have updated the same in the revised version of the manuscript.

**Reviewers’ comments:**

Dear reviewers,

Thanks for considering our manuscript on “Effects of active and passive enrichment regime on fecal glucocorticoid metabolite levels in captive Indian leopards (*Panthera pardus fusca*)”, and appreciating our work as being “robust”, “well conceived” and “interesting”. We greatly appreciate the feedback from all the three reviewers and have addressed the concerns in the revised version of the manuscript. In this document, we have provided specific responses to each of the concerns raised by all the three reviewers.

Comments from reviewer #1

*The authors have provided a robust study that provides valuable information related to the housing and welfare of a cryptic and solitary carnivore in captivity. The manuscript meets the requirements for acceptance however I would encourage the authors to obtain assistance from a native English speaker to assist with grammar and sentence structure. Although the information is important it could be more clearly communicated. I therefore recommend acceptance of the manuscript for publication after major revision.*

Response- Thank you for appreciating our work and considering our manuscript worthy of publishing. We have now substantially revised the manuscript to make the language more clear and succinct.

*Abstract*

1. *Line 12 – improves the*

Response: Yes, we agree with the reviewer and have now changed the same in (line #18) the revised version of the manuscript.

2. *Line 13 – first mention of species in the Abstract should have (scientific name)*

Response: Yes, we agree with the reviewer and have now added the scientific name in the revised version of the manuscript (line #19).

3. *Line 14 – clarify interacting and non-interacting – are zoo keepers interacting with animals or are animals interacting or not interacting with certain aspects of their environment/enrichment mechanisms – perhaps use the term active and passive*
   Response: Yes, we agree with the reviewer and have now removed the “interacting” term and have only used “active” and “passive” in the revised version of the manuscript (line #20).
4. *Lines 16-20 – categories should be more specific – the difference between large, medium and small cage size provides no distinction in cage size for the reader*
   Response: We agree with the reviewer and thanks for your comment. Instead of qualitative terms, we have now added the specific values for cage sizes in the revised version of the manuscript (line #22, 24-25).
5. *Line 28-29 – The authors need to clarify what policies this information will inform – do you refer to the management of captive, free-ranging leopards or both??*
   Response: Yes, we agree with the reviewer and have referred to “captive” only in the revised version of the manuscript (line #34). The inclusion of samples from free ranging leopards was more for technical validation of the hormone assay.
6. *Introduction
   I found the introduction difficult to get through, its also very long – with a bit of restructuring, the authors could build a clear and solid foundation that introduces the importance of their work. I would suggest restructuring the introduction and possibly involving an English speaker to assist with sentence structure, grammar and word order.*

*First introduce the importance of zoos for maintaining genetic integrity of populations and behavioural studies - Focus on India and your species of interest – move lines 118-139 up to the beginning of the intro
Then deal with the difficulties related to the management of zoo animals of different species – which can result in stereotypical behaviour and other negative effects - Then discuss stereotypical behaviours and results of confinement – introduce the concept of enrichment through different mechanisms – food, size of enclosure and define and reference active (Line 58-59) or passive (Line 67-69) enrichments. Introduce the concept of stress and go into some detail related to stress physiology, which can reduce physiological health, cause reproductive issues or welfare issues in captive settings. Then highlight why it is important to be able to monitor stressors in the most effective, non-invasive and standardized way in this species to ensure optimal health of captive individuals and point out the focus of your study and how it bridges the gaps in our knowledge related to your specific species and their management in zoos in terms of monitoring stress.*
Response: We appreciate the reviewer’s suggestion, and after reassessing the introduction, we thought that the suggestion made by the reviewer is very similar to the flow that we have maintained in this section. We first talk about ex-situ conservation, for example, zoos, and then provide details on how enrichment (both active and passive) is important for the well being of captive animals to reduce stereotypic behaviors. Then we introduce the concept of stress and stressor, and how hormone monitoring through non-invasive sampling can help assess the well being of zoo animals. We provide specific examples of large vertebrates kept in isolation under captivity. Finally, we talk about India and the model species for the manuscript. We believe, keeping in mind the broad readership of the journal, the importance of enrichment for zoo animals and use of non-invasive hormone monitoring is of larger importance, globally. India and the target species are presented as the study site/model organism for the manuscript and thus, we would like focus on India (and the leopards) during the later part of the introduction. However, with inputs from all the reviewers, we hope to have made the language more clear and concise.

7. *Line 50 – topmost is not a word*
   Response: Thank you for your suggestion, and we have now revised accordingly (line #48).
8. *Line 57 – the authors state that studies have shown – but don’t reference these studies also no references are given for definitions of active and passive enrichment within the study setting*
   Response: Thank you for your suggestion, and we have now added a citation (#7 in the revised version of the manuscript (line #58).
9. *Line 60 – I do not understand the term “a study in literature” – are the authors referring to a literature review or are they referring to the fact that they did a literature search*

Response: Apologies for the confusion, we refer to a particular study and have	revised accordingly (line #58).

10. *Line 61 refers to ‘proper housing’ - for readers outside of a captive zoo setting, this provides no useful information – be specific in your description of what constitutes proper housing*

Response: Thanks for your comment, we agree and have now removed the word	proper and defined structural enrichment as housing mimicking the natural	conditions of the target species. Please refer to line #59-60 in the revised version of the	manuscript.

11. *Line 62 refers to studies conducted on various species but no references have been given for these studies*

Response: We would like to point out that the original reference [#8] by Carlson	and Shepherdson is a review paper that includes findings on multiple species.

12. *Line 62 and 75 no scientific names of species mentioned are included in the introduction*
    Response: Thank you for your comments. We have added the scientific names of all the species in the introduction section of the revised manuscript (line#60-76).
13. *Line 63-67 is repetitive – “Bears had higher  locomotor and exploratory behaviours when food was provided in different ways, such as in a log filled with honey and by hiding food throughout the exhibit. Multiple ways of food  presentation helped bears to reduce stereotypic pacing from 125min/day to 20min/day, and  increased the rate of their exploratory behaviours [6].” Reword to When food presentation was varied, such as in a log filled with honey and by hiding food throughout the exhibit, bears reduced stereotypic pacing from 125min/day to 20min/day, and increased the rate of their exploratory behaviours [6].*
    Response: Thank you for your suggestion, we have now reworded the sentence in the revised version of the manuscript (line #61-64).
14. *Line 81 “Long-term physiological stress is highly detrimental to zoo animals” it is also detrimental to free-ranging animals – I would focus the attention to zoo animals but not restrict your comments to captive individuals only. You also have no references for this and below statements related to the negative physiological effects of stress.*

Response: Thank you for your comments. We have now added the word “free-	ranging” to the revised version of the manuscript (line #81). We have also added	citations (#11) to support statement (line #80-83).

15. *Line 84 – definition of stress should be referenced
    Distinctions between the stressor and the stress response should be more clearly defined that include physiological and psychological aspects.*
    Response: We have now revised this part in the manuscript, please refer to line #80-83. We have also added citations (#12) to support statement (line #84-85).
16. *Line 85 – “A series of physiological events take place to restore this disruption is defined as the stress response” This does not make sense, the authors are saying that disruption (i.e. stress) is restored by physiological events – I think they mean that homeostasis is restored through the stress response.*
    Response: Thank you for your comment. We have now revised the same (line #85-86).
17. *Line 86-91 – this could be more clearly worded to describe the differences in extrinsic and intrinsic stressors. Utilize terminology suitable for animals – aggression not anger or refer to in humans or in captive animals for these stressors.*
    Response: Thank you for your suggestions. We have now completely revised this part to simplify the language. Please refer to line#89-90 in the revised version of the manuscript.
18. *Lines 91-95 – “Under stressful conditions, stress hormones are released as a biological response   helping to cope with “fight or flee” situations. Most kinds of stressors trigger the release of   glucocorticoid (GC) hormones [10], and GCs are known to increase blood glucose levels,  suppress immune system to help maintain homeostasis, and support metabolism of fats,   proteins and carbohydrates [11,12] to fight against the stressful situations.  ” I do not think this is an adequate description of the stress response – the authors do not clearly describe the physiological processes of the stress response, where GC come from, why they can provide a measure of stress or how they can be measured or that faeces is used in this study because it meets all of the criteria for non-invasive assessment.*
    Response: We have now completed rephrased this section based on the comments of both reviewer #1 and #2, and have added new citations (#10-12), as well. Please refer to line#84-96 in the revised version of the manuscript.
19. *Materials and Methods: This section is quite complicated to read and should be revised to more clearly outline the differences between the sites as well as the categories and number of animals sampled at each site.
    I would suggest splitting the section into two section that deal with Captive and Free ranging or into three sections that deal with Captive and Free-ranging and then Sample collection – Either provide a summary of each site in words then add info (number of categories, number of animals, number of samples, etc.) in a table or provide details in text – there is no need to repeat information or leave information out. If you provide details in the text, make it easier for the reader to go back and check number of categories at a site, or number of samples from an animal. I would start the captive section by describing the overall environmental conditions of the area (as zoos are quite close together) this will help to put any environmental differences between free-ranging and captive animals in context for the reader. Give the same information for each facility – currently you provide size of Kankaria zoo but not number of animals housed but for Sayajibaug zoo you give number of animals and species – Be consistent and be specific.
    Then you provide information on each site specifically – I would keep things simple here. e.g. Leopards housed in both facilities are adults. In Kankaria/Kamala Nehru zoo, Ahmedabad, Gujarat indoor and outdoor housing is provided while at the Sayajibaug zoo only outdoor housing is offered. Provide further details in a comparative way regarding cage size, enrichments etc.*

*Then you describe sample collection methods within each setting – Currently (line 196) you provide total number of samples but do not state from which facilities these samples came from. I would revise Supplementary Table 1 and include it in your methods – or include this information in the text to make it easier for the reader to follow.*

Response: We apologize for the confusion and have now completely revised the materials and methods section of the revised manuscript (line#151-250). As per the suggestion of the reviewer, we have divided this section into two parts, captive and free ranging. We have also revised Tables 1 and 2. We have removed the supplementary table 1 and have included that information in the text, please refer line#187-195 in the revised version of the manuscript.

20. *The authors state that only fresh faecal sample collection from animals in the wild occurred and that determination of freshness was based on moisture content, smell and state of decomposition. If you do not observe the animal defecating, how did you determine state of decomposition? The moisture content, smell and state of decomposition are all determined by the last meal – if the meal consisted of an old carcass, sinew and bone – moisture content, smell and visual content of the faeces will differ markedly to faeces resulting from a blood rich or flesh meal. The authors should consider rewording this to show that samples were considered fresh if “list your criteria” i.e. warm to the touch, fresh tracks visible, <12 hours etc. I do not see any mention of why you use wet weight for some samples and dry weight for others – perhaps you can provide some info on this in your methods??*
    Response: We appreciate the reviewer’s insightful suggestions. Sampling from free-ranging leopards was done in a conflict area with the help of forest department and thus, daily tracking and monitoring of the area was done over the entire sampling period. This was a part of the larger project on monitoring conflict situation in the villages. As a part of this larger project, we encountered fresh scats that were always accompanied by recent activity of the leopards. On a few occasions (for 3 out of 12 samples), there was a direct sighting of the sampled individual. Further, bacterial decomposition, and presence of insects indicate an old scat, which were avoided during our sampling from free-ranging leopards. We have revised this part accordingly, please refer to line#223-225 in the revised version of the manuscript. However, we would like to clarify one major concern that the reviewer raised here. We have analyzed all the samples using only dry weight (DW), the use of wet weight has several issues pertaining to the dietary differences and seasonal differences in water intake, and we have not used wet weight for any of our analyses.
21. *Statistical analysis: You do not include seasonal aspects as one of your stated aims of the project in your introduction or in your data collection sections but you mention it in the statistical analysis and results section – I would suggest making a clear link between possible influences of season on animal welfare in captive individuals and stress levels if this is something you are going to address in your results
    You could also add a single sentence in your statistical analysis section indicating when fGCM are reported as micrograms /gram dry weight (DW) or wet weight (WW) instead of repeating this in your reporting of results for each variable.*
    Response: We thank the reviewer for the suggestion. However, we did not see a clear impact of seasons across the categories. The impact was entangled with individual variations and type of categories. For example, there were significant differences in fGCM levels between summer and winter seasons for leopards in category ‘A’, however, there were no significant differences (P>0.05) in fGCM levels between summer and winter seasons for both categories ‘B’ and ‘C’. For these reasons, we would like to focus on active and passive enrichment only, and not on seasons.

We have included a line (line#279) in the statistical analyses section that all the values of fGCM are expressed in ug/g dry weight of feces. We would like to point out that all samples were dried and expressed in dry weight only.

22. *Results: fGCM profile of captive leopards. If you add a sentence to your statistical analysis for reporting WW and DW – you do not need to state it after reporting each concentration. Your sample size is quite small, how do you account for this limitation in your statistical approach?*
    Response: Thank you for your comment. We have included a line (line#279) in the statistical analyses section that all the values of fGCM are expressed in ug/g dry weight of feces. We would like to point out that all samples were dried and expressed in dry weight only. We appreciate the reviewer’s comment on smaller sample sizes. However, our sample size is comparable to similar studies conducted on large vertebrates in zoos or captive conditions. For example, Vaz et al. Prevalence and determinants of stereotypic behaviours and physiological stress among tigers and leopards in Indian zoos. PLoS One. 2017 had a sample size of 21 scats from 9 individuals, and Crossey *et al.* Using faecal glucocorticoid metabolite analyses to elucidate stressors of African wild dogs *Lycaon pictus* from South Africa, Wildlife Biology. 2020 had a sample size of 47 scats from 35 individuals.
23. *Discussion: Throughout the discussion you refer back to the different categories, it may be easier for the reader to follow if you outline the criteria from that category and then add the category in brackets after i.e. active and passive enrichment (Category A).*

Response: Yes, we agree with the reviewer. We have now outlined the criteria from that category and added the different categories (A, B and C) within parentheses, wherever appropriate, within the discussion section of the revised manuscript.

24. *Lines 382 – 392 – the statement that sex-specific differences in cortisol are not prevalent in other carnivores is a bit misleading as you then state in the next sentence that reproductive state in females influences cortisol concentrations in tigers – the same was observed in the study on African leopards – I suggest rephrasing this to more clearly highlight higher cortisol levels in reproductive females.*
    Response: We appreciate the reviewer’s insightful suggestion and we have accordingly revised the sentence, please refer to line#394-396.
25. *Line 387 – The effect of sex type is not correct – change to the effect of sex/gender.*
    Response: Yes, we agree with the reviewer and have now changed it to the effect of sex. Please refer to line #398 in the revised version of the manuscript.
26. *Line 395 – what do the authors mean by season-wise are you referring to per season?*
    Response: Thank you for your comment. We have now clarified this point in the revised version of the manuscript (line #405-410).
27. *Be careful of using the phrase “in the current study (Lines 351, 360, 374, 384, 389, 394, 411, 429, 440, 453, 463, 468, 470) and or /our study (349, 357, 378, 393, 419, 449, 455” too often – use only when comparing directly to another study.*
    Response: Thank you for your comment, we have revised the discussion section and incorporated the above suggestion accordingly.
28. *You sometimes refer to stress hormone metabolites (lines 397, 405) and then other times to fGCM concentrations/values (lines 395, 403) – remember your work might be of use to people outside the field of endocrinology so keeping your terminology consistent will help readers follow your story better.*
    Response: We appreciate the reviewer’s suggestion and have accordingly revised, please refer to line#406.
29. *The use of “high” does not convey any quantitative information nor does it contextualise the level – please go through the discussion and reword to be more specific where applicable
    Line 403 – you state high fGCM values of leopards in category B – high in comparison to what? Other categories, free ranging animals - you need to be specific.*

*Line 420 high variation – in comparison to what?*

*Line 422 high levels in comparison to what?*

*Line 425*

*Line 426*

*Line 447*

*Line 450*

Response: Thank you for your comment. In the revised version of the manuscript, we have now included specific comparisons, wherever applicable (line#436-442).

30. *Line 415 “Studies have documented that physiological effect “ should read Studies have documented that the physiological effect or physiological effects.*
    Response: Thank you for your comment. We have now revised it accordingly (line #428).
31. *Line 431 – should read “sound proof glass” not glasses and sentence should be in active voice “i.e. Category ‘A’ was the only regime where passive-enrichment elements, for example, natural sounds, sound proof glass to filter visitor noise …“*
    Response: Yes, we agree with the reviewer and have now revised accordingly (line #445-449).
32. *Lines 457 – 463 very long sentence, try breaking up into two*
    Response: Thank you for your comment. We have now revised it accordingly (line #451-456).

Comments from reviewer #2

*The paper submitted by Panchal et al to PLoS ONE reports fecal glucocorticoid metabolite (fGCM) levels in an exploratory study in captive Indian leopards. Animals were kept under three different housing conditions (categorized A-C), providing different types and degrees of enrichments. The authors utilized the same enzyme immunoassay, which has been previously fully validated for African leopards. Overall fGCM levels were much higher in captive animals compared to wild ones, from which a few samples were also included for comparison. Huge individual variations in fGCM levels in captive animals were found, but also differences between seasons, whereas sex did not affect levels. In addition, cage size influenced levels significantly.
Given the setting of the study (for obvious reasons no experiments could be performed), I think it is not possible to speak about “effects” of enrichment. In addition, in categories “A” and “B” only one sex was present, thus the influence of sex and enrichment in those two settings cannot be disentangled. I found the categories, “active” and “passive” enrichment interesting. However, I wonder, whether cage size is really an active enrichment (e.g. line 404)?
Below please find my specific comments. I thought it is easier for all involved to make my suggestions related to style or wording directly into the word file (track mode for changes).*

Response: We thank the reviewer for appreciating our work, and for including the suggestions in a track change mode. We understand the concern of the reviewer for describing cage size as an active enrichment, but based on our analyses and the type of resources available in each condition, cage size appeared to be the only criteria influencing the measured fGCM levels in the sampled captive leopards. We have responded below to all the specific comments raised by the reviewer.

*Further detailed comments (ordered by appearance in the ms):*

1. *Line 1/2: I suggest rewording: “Fecal glucocorticoid metabolites in captive Indian leopards (Panthera pardus fusca) housed under different enrichment regimes”*
   Response: We appreciate the suggestion and have revised accordingly. However, for better clarity, we have included the phrase “measurement of” in the revised title of the manuscript (line#4-6)
2. *Line 14: A (zoo) animal is not a “system”*
   Response: Yes, we agree with the reviewer and have now changed it to organism (line #20)
3. *Line 21 (and elsewhere): You did not “standardize” a non-invasive method. There is no standard available, all immunoassays give relative concentrations when applied to fecal samples (because there is a mixture of different metabolites present there). However, you need to validate such methods (biochemically, but more important physiologically/biologically).*

Response: We appreciate the suggestion and have revised accordingly. Please	refer to line#26-27.

4. *Lines 63-67: I doubt that those details are necessary to describe here. Please delete.*
   Response: We appreciate the reviewer’s suggestion, however, we would like to retain the details to demonstrate that stressful conditions may trigger stereotypic behaviours in animals, particularly under captive conditions.
5. *Line 92: The classical “fight or flight” reaction is linked to the sympathetic nervous systems, and includes the release of catecholamines.*
   Response: Thank you for the comment, we have now removed this statement and revised this paragraph based on the comments of the Reviewer#1.
6. *Line 103: I suggest citing a review here, at least instead of the primate paper (or why is this cited, and not other ones?)*
   Response: We agree with the reviewer and a citation for a review paper (citation #9) has been included in line 72.
7. *Lines 127/128: Add the “metabolites”. Even when the original paper has it wrong, there are no glucocorticoids in the feces, only their metabolites.*
   Response: Thank you for the suggestion. We have revised it accordingly (line #128).
8. *Table 1: I think it would be better to move it to the supplement. Besides, the column “Breeding history does not give any additional information and should be deleted (also the “Remarks”). “Gender” should be replaced by “Sex” – it is about the biological term here.*

Response: Thank you for your comments. Based on the suggestions by reviewer#1, we have revised this section (line#187-195). We have included the original Table 1, as Table 2 in the revised version of the manuscript. We would like to retain the information on individual leopards, to provide the details on capture date and duration in captivity. However, we have replaced, ‘Gender’ term with ‘Sex’, and have deleted the breeding history column.

9. *Table 2: You give the cage side in m3, but if no details about “length*breadth*height” are given, the latter can be deleted.*
   Response: Thank you for the suggestion. We have revised it accordingly (table-1).
10. *Lines 204 and 218: You state that four districts were chosen, but then samples were only collected from three?*

Response: We appreciate the reviewer’s suggestion but we had surveyed all the	four districts, as they support a large population of leopards within the state of	Gujarat. However, during fieldwork, we found fresh scat samples only from three	districts. We have now clarified this point in line#219-222.

11. *Line 215: What accounts for 7.4%, unclear to me?*
    Response: Thank you for the suggestion. We have revised the sentence to provide better clarity (line #217-218).
12. *Lines 237-252: This section needs drastic revision. I have made a suggestion directly in the docx-file. You need to cite the original paper, where the EIA was first described (Touma et al., 2003). 5α-pregnane-3β,11β,21-triol-20-one is the standard of this EIA. However, as this is a corticosterone metabolite, but cortisol is the dominant GC in carnivores, it is most likely not present in the feces. Still, because the EIA picks up metabolites with a 5α-3β,11β-diol structure, which can also be derived from cortisol (respective cross-reactions have only recently been described in Santamaria et al., 2021), this EIA has been proven suited in African leopards [9].*

Response: We thank the reviewer for such detailed inputs; we have revised this	section accordingly, and have added the suggested citations (line#244).

13. *Line 259: Well, category and sex were not independent, because category A had only females and “B” only males.*

Response: We agree with the reviewer, and since sex was not giving any significant results, we have removed the results on mixed effect analyses that included the sex category, and revised the section accordingly. Please refer to line#257-259 in the revised version of the manuscript.

14. *Results: Overall, I think it is more adequate to present levels as median with range (min-max), and show them in respective figure 2 as boxplot graphs. The variation was so huge (sometimes the SE is greater than the mean) and thus data not normally distributed, or?*

Response: We agree with the reviewer, and have now added an additional figure (Figure 3) as boxplot representing median values. However, we would like to mention that log transformation of fGCM values resulted in normality of the data, and thus, we have mentioned Mean±SE all throughout the manuscript.

15. *Line 287: mean±SE?*

Response: Based on the suggestion of reviewer #1, we have added a line (line#280) under statistical analyses section that all values are represented as Mean±SE, and in ug/g/dry weight of feces, and just present values in the results without repeating the units and the terms (Mean±SE).

16. *Line 287 onwards: “μg/g dry wt of feces” – I think you can state somewhere that fGCM levels are expressed as μg/g per dry weight of the feces and then reduce that to “μg/g feces” throughout.*
    Response: Thank you for the suggestion. Based on the suggestion of reviewer #1, we have now added a line (line#280) under statistical analyses section that all values are represented in ug/g dry weight of feces, and have removed all the repetitions throughout the manuscript.
17. *Line 296 – Figure 2: As the SE is huge (sometimes more than the mean), I think it’s better to give boxplot graphs of the values than presenting mean±SE.*

Response: We agree with the reviewer, and have now added an additional figure (Figure 3) as boxplot representing median values. However, we would like to mention that log transformation of fGCM values resulted in normality of the data (line#259-260), and thus, we have mentioned Mean±SE all throughout the manuscript. *Line 300: For reason outlined above (“effects”), I suggest to delete this heading (it should not have the same level as the one above (line 286)*
Response: Thank you for the suggestion. We have revised accordingly, please refer to line#312-313 in the revised version of the manuscript.

18. *Table 4: a p value of 0.0000 is impossible, needs to be <0.0001.*
    Response: Apologies for the typo, we have corrected it.
19. *Lines 344-347: Move this to the supplements. It is only a small technical details and not of general interest (or importance).*
    Response: Thank you for the suggestion. However, keeping in mind the technical details required to support the assay method, we would like to retain this part in the main manuscript itself.
20. *Lines 349-359: Vaz et al (2017) utilized a corticosterone immunoassay. However, this does not mean that they measured corticosterone in the feces (see e.g. Palme, 2019 for a more detailed explanation). They did not characterize the immunoreactive compounds in the fecal samples of Indian leopards. Because glucocorticoids are heavily metabolized prior to excretion and also (as you correctly stated) cortisol and not corticosterone is the predominant GC, it is very likely that their assay picks up (cortisol) metabolites. As cross-reactivity with those metabolites is expected to be low, this perfectly explains the lower fGCM levels reported in [21]. I started to rewrite this paragraph, but I think more needs to be done here. In addition, the assay of Vaz et al. was not validated for leopards, which also warrants mentioning somewhere.*
    Response: Thank you for your comment. We have modified this section and added specific comments on the study done by Vaz *et al*. We have also mentioned that the assay used in the study by Vaz *et al*. was not validated for leopards (line#362-370).
21. *Line 355: I suggest to delete [32, 33], the first does not seem suited at all, and the second is included in the review [34].*
    Response: Thank you for the suggestion. We have accepted the change mentioned by the reviewer in revised version of the manuscript (line #366).
22. *Line 361: Actually, 5α-pregnane-3β,11β,21-triol-20-one is a corticosterone metabolite (the 17α-OH, the only difference between cortisol and corticosterone, is missing). However, because the immunogen for the antibody, and the label of this EIA have been coupled at position C20, the assay cross-reacts with both, cortisol and corticosterone metabolites. This was recently proven (see Santamaria et al., 2021).*

Response: Thank you for the suggestion. We have accepted the change mentioned	in track mode (line #372-373)*.*

23. *Line 387: “very variable”: Are there studies available, which report sex differences in a carnivore species? Otherwise this sentence does not make sense.*
    Response: We agree with the reviewer, and have revised accordingly (line#395-397).
24. *Lines 423/424: This sentence sounds odd. Please reword.*
    Response: We appreciate the reviewer’s suggestion and based on the suggestions of reviewer#1, this section has been revised and the sentence has been removed.
25. *Lines 438-439: I would be very cautiously in drawing conclusions here. You did not perform experiments, just compared different housing situations. To answer such questions I think it would be necessary to plan longitudinal experiments with the same animals and provide different enrichments.*
    Response: Thank you for the suggestion. Based on the suggestions, we have revised the conclusions, please refer to line#451-456.
26. *Line 445: So the two old animals were no longer present in summer?*
    Response: Both the old animals were present during summer. We sampled during the summer months of 2019, and winter months of 2020. They died post-winter, we have added this information in the revised version of the manuscript (line#461-462).
27. *Lines 457-463: Attention! Suggesting to include a huge number of covariates will decrease statistical power, which will result in more negative findings (no effect found), when sample numbers are not increased at the same time. Thus, I think the first demand should be to increase the number of individuals studied and the number of samples collected!*

Response: We agree with the reviewer and have accordingly modified this	section. Please refer to line#476-478 in the revised version of the manuscript.

28. *There is one further study published, which investigated fGCM levels in a single captive Afghan and black leopard each, before, during, and after the period of exhibit construction (Chosy et al., 2014). The authors used a corticosterone and a cortisol EIA, respectively, and found higher levels during construction (up to ~1 µg/g feces). It may be worth including this paper in the discussion.*
    Response: We agree with the reviewer and have included the suggested citation in line#100 in the revised version of the manuscript.
29. *References: The references need careful revision (e.g. capitalized names in 5; first names spelled out in 41) – why are internet links (doi) given for some papers, but not others? Latin names should be in italics, etc…*
    Response: Thank you for your comment. We now have revised the references accordingly.
30. *Figure 1: The resolution of my copy was rather low (but I hope the original one is better), thus it was hard to get the details.*
    Response: Thank you for your comment. We have now increased resolution in the revised version.
31. *Figure 2: I suggest presenting the levels as boxplot graphs (because variation was so huge; and data not normally distributed, or?). Comparing levels here, with data given in the “Results” sections (e.g. line 291 for the winter in “A”: 25.11±29.39 µg/g) make me wonder, why they do not match (the error bar should exceed the box)? Y-axes legend: “fGCMs (µg/g dry feces)”*
    Response: Thank you for your comment. We apologize that we had added the SD values for fGCM levels in the text, but SEM in the figure. However, we have corrected it now. Based on the suggestion of reviewer#1, we have added SEM values all throughout, and have mentioned that in line#280 in the revised version of the manuscript. We have added a new figure (Figure 3) representing median fGCM values of leopards for all categories for both winter and summer seasons.
32. *Figure 3: It would be easier to orient oneself, when different colours and also open symbols are used. Also may be better to give median levels, instead of mean. The y-axes should then read: “median fGCMs (µg/g feces)”*
    Response: Yes, we agree with the reviewer. We have now included median values in the new Figure 4 and have used colors to represent different individuals. Please refer to Figure 4 in the revised version of the manuscript.
33. *Figure 4: As outlined above, I suggest moving it to the supplementary material. And it’s not the sample that is “wild” or “captive” ϑ.*
    Response: Thank you for your comment. As a technical detail to support the data for the fGCM assay, we would like to keep this in the main manuscript. However, we have changed the legend to “captivity” and “free ranging”. Please refer to Figure 5 in the revised version of the manuscript.
34. *Supplemental Information (for review purpose only): I encourage the authors to make that information available in a supplement (especially the number of samples is interesting to know). Also, it may be worth noting (remarks) that Vaz et al did not validate their EIA for use in Indian leopards.*
    Response: Thank you for your comment. Based on the suggestion of reviewer#1, we have added the information of Supplemental Table 1 in the ‘materials and method’ section of the manuscript (line#187-195). Supplemental table 2 is revised as Supplemental Table 1 for the revised manuscript. We have now added a sentence (line#462-464) in the revised version of the manuscript on Vaz et al., stating that they did not perform any validation of the assay for the Indian leopards.

*Above cited references:*

*Chosy, J., Wilson, M., Santymire, R. (2014): Behavioral and physiological responses in felids to exhibit construction. Zoo Biol. 33, 267-274. [https://doi.org/10.1002/zoo.21142](https://doi.org/10.1002/zoo.21142)
Palme, R. (2019): Non-invasive measurement of glucocorticoids: advances and problems. Physiol. Behav. 199, 229-243. [https://doi.org/10.1016/j.physbeh.2018.11.021](https://doi.org/10.1016/j.physbeh.2018.11.021)
Santamaria, F., Barlow, CK., Schlagloth, R., Schittenhelm, RB., Palme, R., Henning, J. (2021): Identification of koala (Phascolarctos cinereus) faecal cortisol metabolites using liquid chromatography-mass spectrometry and enzyme immunoassays. Metabolites 11, 393. [https://doi.org/10.3390/metabo11060393](https://doi.org/10.3390/metabo11060393)
Touma, C., Sachser, N., Möstl, E., Palme, R. (2003): Effect of sex and time of day on metabolism and excretion of corticosterone in urine and feces of mice. Gen. Comp. Endocrinol. 130, 267-278. [https://doi.org/10.1016/s0016-6480(02)00620-2](https://doi.org/10.1016/s0016-6480(02)00620-2)*

Response: Thank you for providing the details of the citations. We have added all the suggested citations (#20, 33, 34 and 36) in the revised version of the manuscript.

Comments from reviewer #3

*The manuscript is scientifically well-conceived and experimental design strategy to examine the environmental enrichment measures was good. Measures of fGCM as a read out to assess the physiological well-being is interesting. The study outcomes provide improvising opportunities in the zoo management practices and findings are important in the context of strategic development of captive management of big cats, Indian leopards, with implications in their conservation.*

*In the manuscript titled, “Effect of active and passive enrichment regimes on fecal glucocorticoid metabolite (fGCM) levels in captive Indian leopards”, authors have measured fGCM levels of captive Indian leopards, housed in three types of housing environments with varied enrichments and, showed that fGCM levels in leopards housed in active enrichment type with small or medium size cages without or with air coolers were significantly different and that housing animals with active enrichment element influence physiological well-being of captive leopards. A number of appropriate statistical analyse were performed to validate the data and conclusions drawn. The study outcomes provide improvising opportunities in the zoo management practices, focusing on categories of animals being housed, coupled with environment specific enrichment. These findings are important in the context of strategic development of captive management of big cats, Indian leopards, with implications in their conservation.*

Response: Thank you for appreciating our work, and considering our manuscript worthy of publishing. Please find below our responses for each of the comments raised during the review process.

*Comments:*

*1. Abstract section: specify values of fGCM levels in categories B and C (lines 25-26). Also, replace “informing” by “informed” (line 29).*
Response: Thank you for your comment. We have incorporated the suggestion in the revised version of the manuscript (line #34).

*2. It is unclear as to what could have been the age of free-ranging animals (n = 12) while collecting opportunistic scat samples. Time elapsed of scat sampling, post-defecation, of free-ranging animals is unclear. This could be a variable factor, while comparing the fGCMs, unlike fresh samples collected from captive animals in zoos.*
Response: Thank you for your comment. Based on the suggestions by reviewer#1 and 2, we have significantly modified this section in the revised version of the manuscript (line#222-225), and have added details on presence of pugmark as a criterion to collect fresh scat samples (possibly within 2-3 hours of defecations). Sampling from free-ranging leopards was done in a conflict area with the help of forest department and thus, daily tracking and monitoring of the area was done over the entire sampling period. This was a part of the larger project on monitoring conflict situation in the villages. As a part of this larger project, we encountered fresh scats that were always accompanied by recent activity of the leopards. On a few occasions (for 3 out of 12 samples), there was a direct sighting of the sampled individual.

*3. Scat samples of zoo animals were collected in distinctly at two different (summer and winter) seasons. However, it is unclear as to why the scat samples of free ranging animals were collected at different times (Jan-Mar), unlike the month duration for scat sampling of captive animals during two seasons.*
Response: We appreciate the reviewer’s insightful suggestion. Wild sampling was done in a conflict area with the help of forest department and thus, daily tracking and monitoring of the area were conducted. The scat samples from free ranging animals were collected only for validation purpose, to apply the same fGCM assay for both captive and free ranging leopards. We have added a line clarifying the same in the revised version of the manuscript (line#147-149). We agree with the reviewer, and thus, we did not include the fGCM values of free ranging leopards in the mixed effect analyses.

*4. What is the GCM extraction efficiency; any recovery experiment performed with scat samples.*
Response: Thank you for your comment. We used a standard protocol for fGCM extraction that has been widely used in several published work, and is in routine use in our laboratory. The average recovery in our laboratory is 88±1.2% based on spiking with a known concentration of GCM.

*5. Line 247: sensitivity – “0.8 pg” indicated is per well or per ml or per gm dry weight?*
Response: Thank you for your comment. The assay sensitivity was 2.4 ng per gram dry weight, and we have corrected this now in the revised version of the manuscript (line #246).

*6. Line 248: A comma is missing at “respectively”.*
Response: Thank you for your comment. We have revised accordingly (line#247).

*7. The last conclusion-paragraph (lines 453-472) should be concise. Redundant statements (for example, ‘enrichment’ and ‘management programmes/policies’) would need to be eliminated.*
Response: Thank you for your comment. Based on the suggestions of reviewer#1 and #2, we have substantially modified the conclusion paragraph and have removed redundant words, accordingly. Please refer to line#476-478 in the revised version of the manuscript.
