# Supplementary material for: Fecal glucocorticoid metabolite levels in captive Indian leopards (Panthera pardus fusca) housed under three different enrichment regimes
Source: PLoS One. 2022 Sep 9;17(9):e0261796. doi: 10.1371/journal.pone.0261796 (PMC9462577; doi:10.1371/journal.pone.0261796)
Supplement: Attachment — Submitted filename: Response to reviewers.docx [file pone.0261796.s005.docx]

Reviewers’ comments:

Dear reviewers,

Thanks for considering our manuscript. We greatly appreciate the feedback from all the reviewers and have addressed the concerns in the revised version of the manuscript. In this document, we have provided specific responses to each of the concerns raised by the reviewers.

**Comments from reviewer #1**

*Dear Authors,*

*The revised manuscript addresses many of the comments and suggestions set out by the reviewers, however one of the fundamental issues you have failed to address is the standard of writing. Your findings have merit and would be of value to other conservation entities and practitioners for the care and welfare of captive leopards, however you have not devoted the same time and effort into accurately communicating your results in grammatically correct English. This oversight detracts substantially from your study. Even your title is grammatically incorrect and I therefore encourage you once again, as I did in my first review, to seek the assistance of a native English speaker or to make use of the services of a professional copyeditor.*

Response: Thank you for your comment, We have now substanitially revised the manuscript, paying closer attention to the language and writing style, as well. We have used services of a professional copyeditor (who is a native English speaker) affliated to Ahmedabad University to seek assistance regarding the language editing. We would also like to point out that the title of the revised manuscript is in aggrement with Reviewer#2 as per the comments received during round 2. We have highlighted several places in the manuscript (please see the revised manuscript) to indicate changes (only with respect to English and writing style, the scientific information remains unchanged) that were made to incorporate the feedback of the copyeditor.

**Comments from reviewer #2**

*Thanks to the authors for substantially modifying their manuscript. It is much improved now, and I’m happy with it (also their responses to my suggestions). There are only a few, marginal things left, which I kindly ask the authors to correct/modify (see below):*

*General: Although you describe that levels are expressed “μg/g feces” somewhere in the methods section, it is still necessary to have the dimension when you report values somewhere (for the ease of the reader, but also correctness). You may avoid repetition in a sentence where several concentrations are given, but would need it at least once.*

Response: Thank you for appreciating our work. We have now added the term *“μg/g feces”* in the results section, as well, particularly when we report several values of fGCM.

*Line 4: delete “levels” it’s obvious and included in “measurement” – otherwise it should read: “fecal glucocorticoid metabolite levels”.*

Response: Thank you, we have removed the term ‘levels’. Please refer to the revised version of the manuscript.

*Line 96: [11] – unsure, but I think that this article only marginally deals with sample materials. What about replacing it with Sheriff et al., 2011 – there pros and cons of the different sample matrices are discussed in detail. Sheriff, MJ., Dantzer, B., Delehanty, B., Palme, R., Boonstra, R. (2011a): Measuring stress in wildlife: techniques for quantifying glucocorticoids. Oecologia 166, 869-887. [https://doi.org/10.1007/s00442-011-1943-y](https://doi.org/10.1007/s00442-011-1943-y)*
Response: Yes, we agree with the reviewer and have now included the citation (#17) in the revised version of the manuscript (line # 87)

*Line 280: µg!*

Response: Thank you for the comment. We have now added the symbol, μg, throughout the manuscript.

*Line 319: <.0001 add the 0 --> <0.0001*

Response: Yes, we agree with the reviewer and have now added 0.0001 (Table 3).

*Line 347: A total of 12 samples was collected.*

Response: Yes, we agree with the reviewer and have now changed the same in (line # 339) the revised version of the manuscript.

*Line 365: [39] Where do they mention this? I suggest citing [33] here instead.*

Response: Yes, we agree with the reviewer and have now included the citation (Palme 2019, number revised as #34) in the revised version of the manuscript (line # 357)

*Line 372: “were detected”*

Response: Yes, we agree with the reviewer and have now changed the same in (line # 363) the revised version of the manuscript.

*Line 428: effects*

Response: Yes, we agree with the reviewer and have now changed the same in (line # 419) the revised version of the manuscript.

*Line 472: I suggested rewording, but it’s still here: “demonstrate” is too strong, especially as you did not perform any experiments to prove this – your study is only observational.*

Response: We agree with the reviewer, and have replaced it with “showed”, please refer to line # 462 in the revised version of the manuscript.
