# Supplementary material for: Fecal glucocorticoid metabolite levels in captive Indian leopards (Panthera pardus fusca) housed under three different enrichment regimes
Source: PLoS One. 2022 Sep 9;17(9):e0261796. doi: 10.1371/journal.pone.0261796 (PMC9462577; doi:10.1371/journal.pone.0261796)
Supplement: Attachment — Submitted filename: Response to reviewers.docx [file pone.0261796.s006.docx]

Dear editor and the reviewers,

Thanks for considering our manuscript on “Fecal glucocorticoid metabolite levels in captive Indian leopards (*Panthera pardus fusca*) housed under three different enrichment regimes”. We greatly appreciate the feedback received from reviewer#1 and have addressed the concerns in the revised version of the manuscript. In this document, we have provided specific responses to each of the concerns raised by the reviewer.

Comments from reviewer #1

- Although somewhat improved from previous versions, this version still contains grammatical errors and the use of language that is not appropriate for scientific writing. See some examples below. As mentioned in previous revisions of this MS, the information is worthy of publication and is relevant for captive population measurement, however, the information needs to be communicated more clearly.

*Response: We appreciate the reviewer’s remark that the manuscript has improved when compared to the previous versions. As mentioned earlier, we have consulted a copy editor, and the current version includes all the suggestions, including language style and grammar, as recommended by the editor.*

- Line 80: Stressful conditions may also impact animal “emotions” Emotions is an anthropomorphic term – rather use behavior

*Response: Yes, we agree with the reviewer and have now replaced the word (please refer to line #80) in the revised version of the manuscript.*

- Line 81: increased aggression or increased repulsion under such conditions – Can the authors clarify what they are trying to say with this sentence
  If they are referring to animal response related to a stressor and they are referring to behaviour – the authors should use the correct terminology – increased aggression or show of submissive behavior.

*Response: Apologies for the confusion, we are referring to animal response related to a stressor. We agree with the reviewer and have now replaced the term (please refer to line #81) in the revised version of the manuscript.*

- Line 89: poor reproductive performances – change to performance

*Response: Yes, we agree with the reviewer and have now incorporated the suggested correction (please refer to line #89) in the revised version of the manuscript.*

- Lines 93-95: Thus, to improvise ex-situ conservation efforts, enriched habitats are provided to captive animals and their physiological response towards the enrichment can be assessed through measurements of stress hormones, mostly by monitoring levels of GCs. Can the authors clarify what they mean by “improvise ex-situ conservation efforts. My understanding of the research, is that the authors used faecal samples and fGCM measurements to quantify stress in relation to different passive and active enrichments but here the authors talk about measuring stress hormones, mostly by monitoring levels of GCs as if they are measuring the biologically active steroid hormone for glucocorticoid measurement in the blood – The authors should clarify this please

*Response: Dear Reviewer#1, thanks for your concern. You are correct in pointing out that we have measured fGCM in captive leopards to quantify stress in relation to different enrichment regimes. However, line#s93-95 belong to the introduction section, where we talk about the biological or physiological response of a stressor. We have explained (please refer to line#s 82-95), how stressors trigger the release of GCs in the body and how GCs maintain homeostasis. The introduction first talks about GCs to provide the readers a background on understanding the physiological response of an individual to a stressor. Thus, we would like to retain the term “GCs” in line#93-95. After talking about the GCs, we have introduced the term ‘fGCM’ in line#105, and then have talked about our topic of research or the objectives for the current study. We have written in this manner to give the readers the correct flow of information, starting from circulatory levels of GC, then to GC metabolites present in different biological samples, including feces, and finally introducing the objectives of our study. Hope this clarifies the concern raised by reviewer#1.*
